# The role of MRI in perianal fistulizing disease: diagnostic imaging and classification systems to monitor disease activity

**DOI:** 10.1007/s00261-024-04455-w

**Published:** 2024-08-24

**Authors:** Jeanine H. C. Arkenbosch, Oddeke van Ruler, Annemarie C. de Vries, C. Janneke van der Woude, Roy S. Dwarkasing

**Affiliations:** 1 Department of Gastroenterology and Hepatology, Erasmus University Medical Center, Rotterdam, The Netherlands; 2 Department of Radiology and Nuclear Medicine, Erasmus University Medical Center, Rotterdam, The Netherlands; 3 Department of Surgery, IJsselland Hospital, Capelle aan den IJssel, The Netherlands

**Keywords:** Perianal fistula, Crohn’s disease, Diagnostic imaging, Scoring systems, Surgery, MRI, Cryptoglandular fistula

## Abstract

Perianal fistulizing disease, commonly associated with Crohn’s disease, poses significant diagnostic and therapeutic challenges due to its complex anatomy and high recurrence rates. Radiological imaging plays a pivotal role in the accurate diagnosis, classification, and management of this condition. This article reviews the current radiological modalities employed in the evaluation of perianal fistulizing disease, including magnetic resonance imaging (MRI), endoanal ultrasound, and computed tomography (CT). MRI, recognized as the gold standard, offers superior soft tissue contrast and multiplanar capabilities, facilitating detailed assessment of fistula tracts and associated abscesses. CT, although less detailed than MRI, remains valuable in acute settings for detecting abscesses and guiding drainage procedures. This article discusses the advantages and limitations of each modality, highlights the importance of standardized imaging protocols, and underscores the need for interdisciplinary collaboration in the management of perianal fistulizing disease. Future directions include advancements in imaging techniques and the integration of artificial intelligence to enhance diagnostic accuracy and treatment outcomes.

## Introduction

Clinical management of perianal fistula is challenging, for both patients and treating physicians [1]. The anatomy of perianal fistula is often complex with involvement of the anal sphincters and multiple side branches [2, 3]. Adequate imaging with accurate assessment of the main fistula tract, side branches and fistulous abscesses, is essential for clinical management [4].

Magnetic resonance imaging (MRI) is considered the standard imaging modality to objectively diagnose fistulizing disease. MRI classification systems have been developed to classify fistula morphology and disease activity, specifically in Crohn’s disease (CD) related fistulas. These scoring systems are mostly used in research settings, whereas the current clinical application of MRI scoring systems in evaluating treatment response is limited [5].

The aim of this review is to describe the role of imaging in the diagnosis of perianal fistulizing disease. Furthermore strengths and limitations of existing MRI-based classification systems to monitor perianal fistulizing disease activity are discussed.

### Etiology and clinical presentation of perianal fistula

A perianal fistula is an epithelialized tract which typically connects the endoluminal surface of the rectum or anal canal with the perianal or perineal skin [4]. Perianal fistulizing disease is rather common with a reported yearly incidence of 1.2–2.8 per 10,000 inhabitants in Europe [6]. The majority of fistula is of the cryptoglandular type, which are thought to result from idiopathic occlusion of the anal glands followed by secondary inflammation, ulceration and ultimately fistulization with the anorectal lumen. Another common etiology of perianal fistula is CD, with a reported incidence of perianal fistulizing disease of 30–50% in patients with CD [1]. Other disease-specific etiologies for perianal fistula include malignancy, chronic pelvic infection, diverticulitis, and exposure to high-dose pelvic radiotherapy. Rare causes concern actinomycosis, lymphogranuloma and, mostly occurring in developing countries, tuberculosis infection of the pelvic area [7]. With regards to the pathogenesis of perianal fistula, studies have shown that epithelial-mesenchymal transition (EMT) plays a significant role in fistula formation, especially in CD [8]. EMT is a reversible process in which the characteristics of epithelial cells undergo multiple biochemical changes, resulting in a mesenchymal cell phenotype. Fistula formation is likely due to EMT of intestinal fibroblasts impairing the ability of these cells to repair mucosal damage [9].

Patients generally present with a combination of symptoms that may include pain, soiling, and discharge of blood or pus from the external fistula opening or the anus, and sometimes fecal incontinence.

### Diagnostic modalities in perianal fistula disease

#### Cryptoglandular fistulas

With MRI, cryptoglandular fistula extensions, internal and external openings and abscesses can be identified with high sensitivity and specificity, and MRI can be used to direct operative procedures. In unclear cases, CT-fistulography, may offer additional information to MRI on the location of the internal opening, which correlates well with operative findings [10]. In general, superficial abscesses and simple distal fistulas do not require diagnostic imaging to guide treatment in both cryptoglandular and CD related fistulas [11]. In addition, dedicated MRI may reveal clinical occult perianal fistula in up to 39% of patients with chronic anal and perianal pain referred to a tertiary center [11].

CT of the pelvis is less sensitive compared to MRI in identifying fistula tracts. In acute setting, CT-scan is often considered to rule out concomitant abscesses [12]. CT-fistulography, a modality that uses fluoroscopy and contrast material to identify fistula tracts, can be useful in clinical practice but requires experienced radiologists and surgeons to perform the procedure and interpret the images [13]. Its usage is predominantly recommended in preoperative setting for complex fistulas with multiple external openings as a problem solving tool or in patients who cannot undergo MRI.

#### Crohn’s disease

**Fig. 1:**
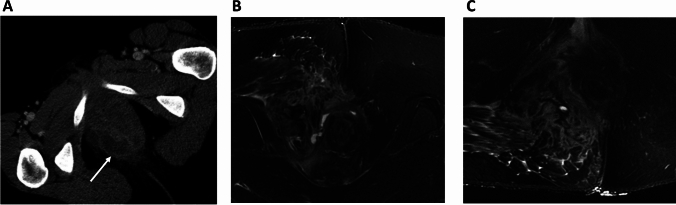
A computed tomography (CT) scan can be used in emergency setting to diagnose pelvic abscesses and to determine the presence and extent of pelvic sepsis. CT-scan of a 37 year old female with Crohn’s disease with clinical complaint of a perianal abscess. **A** CT-scan in emergency setting confirmed perianal abscess (arrow). **B**, **C** Follow-up postoperative MRI one day after surgical treatment. Resolution of the abscess after incision and drainage and small perianal fistula tracts remain visible. Furthermore, MR-images depict tissue inflammation surrounding the fistula tract

CD-related fistulas are more complex than cryptoglandular fistulas as these fistulas often have more side branches, abscesses and extensive tissue inflammation. Unanticipated abscesses, usually in the pelvic region, perianal- or ischiorectal fossa, are frequently found on imaging (Fig. 1) [14]. MRI is considered to be the reference imaging modality for diagnosis and classification of perianal fistula in CD because of its superior soft tissue contrast, multiplanar capability, and lack of ionizing radiation [15, 16]. MRI is better suited as an objective modality to be discussed in a preoperative, multidisciplinary setting. Limitations of MRI [17] include relative high costs and the general contraindications for MRI such as metallic foreign bodies or non- MRI compatible medical devices. In addition, it is of importance to notice that demonstration of fistula resolution on MRI may lag behind clinical healing [18].

CT-scans in patients with CD may provide useful information regarding the presence and extent of pelvic sepsis and abscesses in emergency setting when MRI is not readily available (Fig. 1) [16].

Contrast-enhanced pelvic MRI is the recommended imaging modality for perianal CD by the European Crohn and Colitis Organization to visualize perianal fistula tracts and abscesses [19–21]. Various other imaging modalities for the evaluation of perianal fistula exist.

After exclusion of rectal stenosis, anorectal ultrasound (EUS) offers an affordable and easy to use diagnostic modality in CD related fistulas (accuracy 56–100%) [19]. In experienced hands, the diagnostic accuracy of EUS may be comparable to MRI, especially when combined with hydrogen peroxide contrast enhancement (3D EUAS) [22, 23]. The specificity for the diagnosis is relatively low for both, especially for EUS (43%), compared to MRI (69%) [17]. Therefore, diagnostic imaging with EUS and MRI can be used complementary before surgical exploration [17].

Examination under anesthesia (EUA) plays an important role in the diagnosis and classification of perianal fistula in CD. EUA has a high accuracy for the diagnosis of CD perianal fistula (EUA 90% vs. MRI 76–100%) [15]. When an abscess is suspected, EUA followed by direct treatment with abscess drainage or seton placement is recommended [24]. Furthermore, EUA is associated with a significant interrater variability.

The presence of proctitis is a negative prognostic factor for healing and has therapeutic relevance for treatment options. Therefore, proctosigmoidoscopy is generally recommended in the work-up of CD-related perianal fistulizing disease.

### MRI protocol for systematic assessment of perianal fistula

**Fig. 2:**
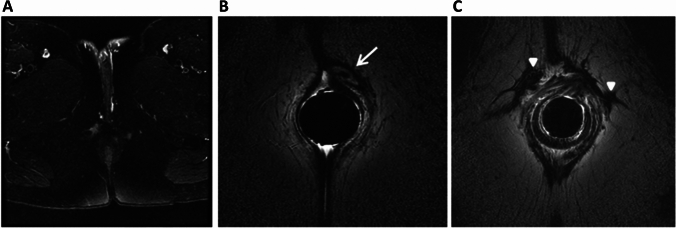
Pelvic Phased array versus endoluminal coils on postoperative MRI after incision and drainage of perianal abscess including seton drain placement. Axial T2-weighted (T2W) with fat suppression (**A**) and without (**B**, **C**). **A** On the external phased array image no evident lesions are seen; conversion to endoanal coil (1.5T) demonstrates a sub-sphincteric fistula with drain in situ (arrow), **B** including the patent ostia of two fistula tracts. **C** Arrowheads point at fibrotic remnants of dried up fistula tracts

For adequate imaging, MRI should be performed with T2-weighted sequences (with and without fat suppression) [25]. The MRI protocol from the European Society of Gastrointestinal and abdominal radiology (ESGAR) [16] recommends field strengths of 1.5 Tesla (T) or 3 T, according to local availability and radiologist preference. Endoluminal receiver coils are considered unnecessary to achieve diagnostic imaging quality [16]. Although these coils may further improve spatial resolution, it goes at the cost of limited field of view and may be associated with patient discomfort (Fig. 2).

**Fig. 3:**
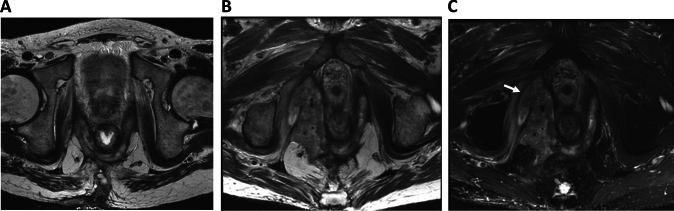
MRI of malignancy complicating perianal fistulizing disease in a patient with Crohn’s disease. **A** Initial T2W MRI; **B**, **C** follow-up T2W MRI (with and without fat suppression). A 42 year old male patient had a history of chronic persistent fistula (**A**) and refrained from treatment for 5 years. On follow-up MRI a mass lesion is visible, arising within the fistula tract and ending into the supralevator space with evident invasion in the surrounding tissue (arrow)

Images in axial, coronal and sagittal plane are required, with alignment of the axial plane perpendicular to the anal canal axis [16]. For at least one acquisition, the supralevatoir and ischioanal compartments should be included to capture possible secondary extensions of the primary fistula tract [16]. Intravenous administration of gadolinium-based contrast agents may allow for better delineation of fistula tracts in CD patients within an inflamed region, including differentiation of the contents of the fistula tracts (liquid, inflammatory or solid (fibrotic) tissue, abscesses). This may also be valuable in patients with chronic persistent fistulas to detect early signs of malignancy complicating fistula disease (Fig. 3). A dosage of 0.1 mmol/kg gadolinium is considered sufficient [26]. A retrospective study demonstrated comparable diagnostic accuracy in contrast versus non-contrast MRI [27]. In general, contrast-enhanced MRI is considered unnecessary in uncomplicated cases.

### Radiological classification systems for perianal fistula

Clinical evaluation combined with MRI can be used to classify a fistula as either simple or complex; thereby guiding treatment decisions and the classification may be indicative for healing rates. The definition of simple or complex fistula varies in the literature. The majority of fistulas are of the simple type [14], which is defined as superficial fistulas, low intersphincteric, or low transsphincteric fistulas involving less than 30% of the distal sphincter complex, often with only one internal opening in the anal canal. Complex fistulas are defined as high transsphincteric fistulas that involve more than 30% of the external sphincter; fistulas with suprasphincteric, extrasphincteric, or horseshoe extensions; multiple side-branches or fistulas associated with radiation injury or malignancy. Complex fistulas may have multiple internal openings in the anorectum and often require multiple surgical procedures aimed at fistula closure [26].

Originally, the Parks classification (a clinical, anatomy based score) and the St James’s University Hospital classification (a MRI based score) were widely applied to classify the extent of perianal CD fistulas and guide surgical decision making (Table 1). More recently, several MRI-based scores ((modified) van Assche Index and the MAGNIFI-CD score amongst others) have been developed to objectify treatment response based on morphology criteria combined with functional MRI features (e.g. signal intensity on T2-weighted imaging) [28, 29].

**Table 1:** Radiological scoring systems used in to classify perianal CD fistulas

	Morphological features	Functional imaging features	Clinical features	Remarks
Park’s (1976) [21]	• Intersphincteric • Transsphincteric • Suprasphincteric • Extrasphincteric	–	Surgical anatomy with external anal sphincter as reference point	Does not correlate with disease severity (e.g. abscess, number of side tracts)
St. James (2000) [30]	• Normal • Simple linear intersphincteric • Intersphincteric with intersphincteric abscess or secondary fistulous tracts • Transsphincteric • Transsphincteric with abscess or secondary tracts within the ischioanal or ischiorectal fossa • Supralevator and translevator	–	Higher grade indicates the need for more complex surgery	• Does not correlate with disease severity (e.g. T2W intensity of lesions) • Uses axial and coronal MR images
Original Van Assche index (2003) [20]	• Number of fistula tracts • Location • Extension • Hyperintensity T2-weighted images • Collections (cavities > 3mm) • Rectal wall involvement	• Hyper intensity on T2 • Rectal wall involvement	Reflects changes in imaging features in response to clinical treatment effect	Does not account for reduced fistula caliber
Modified van Assche (2017) [22]	• Extension • Hyperintensity T2-weighted images • Rectal wall involvement • Inflammatory mass • Dominant feature	• Hyper intensity on T2 • Rectal wall involvement	Decreases significantly in patients responding to anti-TNF[26]	No clear added value of the modified over the original van Assche index [26]
MAGNIFI-CD (2019) [27]	• Number of fistula tracts • Hyperintensity on post-contrast T1W images • Dominant feature • Fistula length • Extension • Inflammatory mass	• Hyper intensity on post-contrast T1W images	–	• Appears to be superior with regards to inter- and intra-rater variability compared to (modified) van Assche Index • Contrast administration needed

**Fig. 4:**
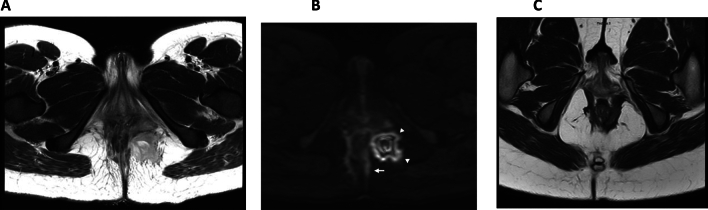
Pre- and postoperative MRI in patient with Crohn’s disease showing clinical and imaging response after surgical fistula closure. **A**, **B** Preoperative T2W- (**A**) and DWI- MRI, b = 1000 s/mm^2^ (**B**) show a transsphincteric fistula (arrow) with presence of a perianal abscess (arrowheads); **C** postoperative T2W MRI showing radiological resolution with absence of fistula activity and abscess (preoperative Original van Assche score of 16 points versus a postoperative Original van Assche score of 0 points)

The van Assche Index was first introduced in 2003 specifically for CD related fistula. This is an MRI-based scoring system describing the location of the fistula tracts as well as the presence of abscesses, proctitis, and T2-weighted signal intensity [20]. The van Assche index was modified in 2017 with addition of the grade of T2-weighted hyperintensity and the presence of infiltrate for a more detailed description (Fig. 4) [30, 31]. As similar radiological characteristics can be found in non-CD perianal fistula, a recent study showed that the modified van Assche index can also be applied in fistulas not related to CD [12].

Both the original and modified van Assche index have the potential to quantitatively monitor disease activity, with a lower score indicating a positive response to therapy [32]. A study demonstrated that a positive clinical effect after anti-TNF treatment for one year was associated with a significant improvement in the van Assche Index, especially with regard to T2 hyperintensity [33]. While the modified van Assche index decreases in the majority of CD patients with perianal fistula who respond to anti-TNF treatment, one third of clinical responders had unaltered scores at follow-up [34]. In addition, correlation between the van Assche index with the clinical score for perianal disease activity (PDAI score) appears to be weak and no correlation with C-reactive protein (CRP), as measurement for inflammation, was found [30]. The role of radiological response to direct clinical management remains unclear, as early clinical healing may not necessarily correspond to obvious changes in MRI features [10, 28]. On the other hand, complete fibrosis of the fistula tract has shown to be a clear predictor for long-term fistula closure [35].

The added value of the modified van Assche index remains uncertain. In terms of inter- and intra-observer variability, no clear benefits seem to be associated with the modified van Assche index. A study by Hindryckx et al. showed an intra-rater variability of 0.81 (95%CI 0.71–0.86) and an inter-rater variability of 0.68 (95%CI 0.56–0.77) for the original van Assche Index versus an intra-rater variability of 0.81 (95%CI 0.74–0.86) and inter-rater variability of 0.67 (95%CI 0.55–0.75) of the modified van Assche index [36].

The relatively new classification score, the MAGNIFI-CD, was introduced in 2019 for perianal fistula in CD patients. The score is based on items selected through expert consensus by four radiologists in a linear regression model using backward elimination [36]. This score includes the number of tracts, hyperintensity on post-contrast images, T1-weighted features such as fistula length, extensions, inflammatory mass and dominant feature. The MAGNIFI-CD, compared to the (modified) van Assche index, appears to have better operating characteristics [4, 36]. Intra- and interobserver variability appears to be better for the MAGNIFI-CD compared to the (modified) van Assche index with intraclass correlation coefficients for MAGNIFI-CD, the modified Van Assche Index, and Van Assche Index of 0.74, 0.67 and 0.68 respectively concerning inter-rater reliability. The degree of fibrosis and MAGNIFI-CD scores are accurate predictors for long-term clinical closure [35].

### Emerging technologies in diagnostic imaging

Qualitative MRI has been increasingly used for monitoring in cryptoglandular and CD related perianal fistula and has shown potential for evaluating response to treatment [18, 30]. The addition of quantitative MRI sequences, such as diffusion-weighted imaging (DWI), dynamic contrast enhancement (DCE) and magnetization transfer, along with T2 relaxometry, to the standard MRI protocols, may offer opportunities to improve diagnostic and monitoring capabilities in cryptoglandular and CD related perianal fistula [30, 37].

DWI is a method of based on the random Brownian motion of water molecules and evaluates the molecular function and micro-architecture of the body [38]. The DWI sequence shows fistula as hyperintense signal abnormality showing restricted diffusion. DWI combined with a mean apparent diffusion coefficient (ADC) calculation, which measures the diffusive movement of water molecules within tissue, has a good performance in differentiating active from inactive fistula [39]. In CD specifically, promising studies have found that both DCE and DWI can be used to assess fistula activity [37, 40], furthermore, magnetization transfer may distinguish inflammatory fistula from more fibrotic fistula [41]. DWI sequences might assist in diagnosing perianal fistula and associated abscesses with confidence [42] by the means of restricted diffusion of water molecules due to viscous pus [43, 44]. Combined DWI-T2W evaluation appears to have a better performance in the detection of fistula than DWI or T2W alone [45]. It should be noted however that DWI sequences have a relative long acquisition time which may increase examination time [45].

T2 relaxometry is a method that uses relaxation times which reflect changes in tissue density or chemical composition; therefore, relaxometry can add sensitivity to conventional MRI scans. A recent cohort study showed that baseline T2 relaxometry, a method that uses changes in tissue density or chemical composition, combined with CRP and clinical symptoms, is a moderate predictor to identify CD patients with perianal fistula who will respond to biologic treatment [43]. In this study, 25 patents with perianal Crohn’s disease underwent quantitative MRI sequences before and 12 week after biological therapy [43]. Contrast-enhanced T1W imaging appears to be comparable to T2W images combined with DWI [45]. A recent study showed that contrast-enhanced endoanal ultrasound may provide an alternative to contrast-enhanced MRI, with similar accuracy in diagnosis of active perianal fistula [46, 47].

The beforementioned MRI classification systems are associated with inter-observer variation and an objective method for fistula volume quantification is lacking. Few, small studies have shown that manual fistula volume measurement is feasible and has a good inter-observer agreement (intra-class correlation 0.95 [95% confidence interval 0.91–0.98] for CD related perianal fistula [32, 33]. Larger prospective studies are needed to determine the correlation between volume changes and clinical outcomes.

Deep learning artificial intelligence (AI) technology is widely applied in the research field of imaging [48]. Studies concerning perianal fistula imaging with deep-learning AI technology are limited. A recent study showed that deep learning-based MRI feature analysis of perianal abscesses and fistulas in Crohn’s disease had a high diagnostic accuracy and played a positive role in improving the accuracy of fistula detection and classification [49]. Another study looked at optimizing CT images of perianal fistula using a convolutional neural network (CNN) algorithm. CT images processed by this CCN algorithm were reported to be clearer, leading to a higher accuracy of preoperative diagnosis [50]. More high quality studies are needed to determine the additional value of deep-learning technology for diagnosis, management and outcome of perianal fistula disease.

## Conclusions and recommendations

**Table:** 

Imaging modalities
- MRI is considered the golden standard imaging modality in perianal fistulizing disease to diagnose perianal fistulizing disease and monitor disease resolution after medical and surgical treatment
- MRI combined with DWI-T2W evaluation appears to have a better performance in the detection of active fistula disease than DWI or T2W alone
- Contrast-enhanced MRI can be considered for chronic persistent CD fistulas to better delineate fistula tracts, abscesses and to identify malignancy
- CT-scan (contrast-enhanced) in CD may provide useful information on the presence and extent of pelvic sepsis and abscessed in emergency setting
- Proctosigmoidoscopy is recommended in the work-up of CD-related perianal fistulizing disease for the assessment of concurrent proctitis

MRI-based scoring systems
- MRI-based diagnostic scoring systems have been introduced for CD related fistulas, with ongoing research to implement these systems for monitoring of cryptoglandular fistulas
- The correlation between clinical treatment response and changes in the van Assche-score seems moderate. Resolution of the fistula tracts on MRI may lag behind early clinical closure
- The modified van Assche index offers no clear added value compared to the original van Assche index
- The MAGNIFI-CD has potentially less intra- and interobserver variability compared to the (modified) van Assche index
- Complete obliteration or fibrosis of the fistula tract on MRI appears to be an adequate marker for long-term fistula closure

Advancements in imaging in perianal fistulizing disease
-EUS in experienced and skilled hands can have equal diagnostic accuracy compared to MRI for the diagnosis of perianal fistula. MRI offers diagnostic images which are better suited for multidisciplinary discussions
- The importance of artificial intelligence in the diagnosis and classification of perianal fistula seems promising but remains to be determined with high quality study designs

## Abbreviations

ADC: Apparent diffusion coefficient
CD: Crohn’s disease
CI: Confidence interval
CNN: Convolutional neural network
CRP: C-reactive protein
DWI: Diffusion-weighted imaging
MRI: Magnetic resonance imaging
DCE: Dynamic contrast enhancement
EAU: Endoscopic anorectal ultrasound
EUA: Examination under anesthetic
EUS: Endoscopic anorectal ultrasound
MT: Magnetization transfer
